# Women’s enlightenment and early antenatal care initiation are determining factors for the use of eight or more antenatal visits in Benin: further analysis of the Demographic and Health Survey

**DOI:** 10.1186/s42506-020-00041-2

**Published:** 2020-06-03

**Authors:** Michael Ekholuenetale, Chimezie Igwegbe Nzoputam, Amadou Barrow, Adeyinka Onikan

**Affiliations:** 1 Department of Epidemiology and Medical Statistics, Faculty of Public Health, College of Medicine, University of Ibadan, Ibadan, Nigeria; 2 Center of Excellence in Reproductive Health Innovation (CERHI), College of Medical Sciences, University of Benin, Benin City, Nigeria; 3 Department of Public & Environmental Health, School of Medicine & Allied Health Sciences, University of The Gambia, Serekunda, Gambia; 4 Project Management Unit, Management Sciences for Health, Abuja, Nigeria

**Keywords:** ANC, Sub-Saharan Africa, Benin, Maternal health, Women

## Abstract

**Background:**

Within the continuum of reproductive health care, antenatal care (ANC) provides a platform for vital health care functions, such as disease prevention, health promotion, screening, and diagnosis. It has been widely confirmed that by implementing appropriate evidence-based practices, ANC can save lives. Previous studies investigated the utilization of ANC based on the four visits model. The new guidelines set by the World Health Organization 2016 recommended increasing contacts with health providers from four to eight contacts. The present study aims to determine the frequency, determinants, and socioeconomic inequalities of ANC utilization based on the eight or more contacts in Benin. This will provide information for policy makers to improve ANC utilization.

**Methods:**

We used a population-based cross-sectional data from Benin Demographic and Health Survey (BDHS)—2017–2018. The outcome variable considered for this study was coverage of ≥ 8 ANC contacts. About 1094 women of reproductive age who became pregnant after the new guideline of ≥ 8 ANC contacts was endorsed were included in this study. The determinants for ≥ 8 ANC contacts were measured using multivariable logistic regression. Concentration (Conc.) Index and Lorenz curves were used to estimate the socioeconomic inequalities of ≥ 8 ANC contacts. The level of significance was set at *P* < 0.05.

**Results:**

The coverage of ≥ 8 ANC contacts was 8.0%; 95%CI 6.5%, 9.7%. The results of timing of antenatal care initiation showed that women who had late booking (after 1st trimester) had 97% reduction in ≥ 8 ANC contacts compared with women who initiated ANC contacts within the first trimester (adjusted odds ratio (AOR) = 0.03; 95% CI 0.00, 0.21). In addition, women with medium or high enlightenment were 4.55 and 5.49 as more likely to have ≥ 8 ANC contacts, compared with women having low enlightenment (AOR = 4.55; 95% CI 1.41, 14.69 and AOR = 5.49; 95% CI 1.77, 17.00, respectively). Conc. Index for the household wealth-related factor was 0.33; *p* < 0.001 for urban women and 0.37; *p* < 0.001 for the total sample. Similarly, Conc. Index for maternal education was 0.18; *p* = 0.006 for urban women and 0.21; *p* < 0.001 for the total sample.

**Conclusion:**

Secondary analysis of the BDHS showed low coverage of ≥ 8 ANC contacts in Benin. In addition, women’s enlightenment, early ANC initiation, and socioeconomic inequalities determined the coverage of ≥ 8 ANC contacts. The findings bring to limelight the need to enhance women’s enlightenment through formal education, exposure to mass media, and other channels of behavior change communication. Health care programs which encourage early antenatal care initiation should be designed or strengthened to enhance the coverage of ANC contacts in Benin.

## Introduction

Globally, pregnancy-related complications contribute to over 50% of deaths among women yearly. According to the World Health Organization (WHO), approximately 90–95% of these mortalities come from resource-poor countries [1]. In 2016, it was reported that the proportion of maternal mortality ratio between resource-constrained settings and developed world was 239 and 12 per 100,000 live births respectively [2]. The staggering reports of maternal deaths in resource-constrained settings have drawn global attention for urgent intervention, as most of the deaths could be prevented through skilled birth attendance and increased ANC contacts. Increased ANC contacts are recommended to reduce maternal deaths by up to 8 per 1000 live births [3]. It can facilitate the uptake of preventive measures, timely detection of danger signs, reduction in complications, and address health care inequalities including those in remote and marginalized settings [4].

In light of the above, WHO in 2016 unveiled a new antenatal care model by increasing contacts with health providers throughout the pregnancy period from four to eight contacts [3]. The guideline is targeted to respond to the complex nature of the challenges surrounding the practice and delivery of ANC within the health systems, and to prioritize woman-centered care and well-being and not to prevent deaths and complications only. Evidence showed that a higher frequency of ANC contacts with the health care providers could lead to a significant reduction in adverse health conditions [3, 4]. The increased opportunities to detect and manage potential problems could enhance services such as counseling on healthy diet, optimal nutrition, blood tests, uptake of intermittent preventive treatment in pregnancy, and tetanus vaccination [3].

Evidence-based data has pointed that the focused antenatal care (FANC) model developed in the 1990s was linked with persistent perinatal and maternal deaths [5]. Meanwhile, an analysis of the WHO ANC trial showed that persistence in perinatal mortality was more likely to be due to increased stillbirths [6]. The contacts during the third trimester are at critical time points that may allow assessment of well-being and interventions to reduce stillbirths. Moreover, evidence suggests that an increase in the number of ANC contacts seems to be associated with an increase in maternal satisfaction and well-being compared with fewer ANC contacts [7].

Numerous factors influence the uptake of optimal ANC contacts. Empirical studies have shown that maternal age [4], educational level [8], planned pregnancies [9], and timing of first ANC visit [10] are factors associated with adequate ANC visits. In addition, exposure to mass media, family income, and accessibility of the obstetric service are also associated with increased utilization of antenatal care [9, 11, 12].

Benin, like several sub-Saharan Africa (SSA) countries, has an unfair share of adverse maternal health outcomes, including pregnancy-related complications and deaths. Worst still, the majority of maternal health indicators such as ANC visits, institutional delivery, post-natal care, and contraceptive use have not improved beyond several SSA countries [13]. However, no study so far could be traced that examined the coverage and factors associated with the new WHO ANC guideline of minimum 8 ANC contacts in Benin. This study is conducted to explore the coverage, determinants, and the socioeconomic inequalities of ANC utilization based on the eight or more ANC contacts in Benin.

## Materials and methods

### Data source

We utilized a population-based cross-sectional data from Benin Demographic and Health surveys (BDHS). BDHS 2017-18 is the fifth of its kind. About 1094 women of reproductive age who became pregnant after the new guideline of ≥ 8 ANC contacts was endorsed were included in this study. The data is available in the public domain and accessed at http://dhsprogram.com/data/available-datasets.cfm.

### Study design

BDHS used a stratified multi-stage cluster random sampling technique, and data was collected on vital reproductive health issues via structured interviewer-administered questionnaires. BDHS used nationally representative data to collect information on demographic, health, and nutrition indicators. The survey is funded by the United States Agency for International Development (USAID). BDHS 2017-18 utilized households as the sampling unit. Within each sample household, all eligible women were interviewed. Details of DHS data collection procedure have been reported previously [14].

### Study settings

Benin has twelve geographical regions: Alibori, Atacora, Atlantique, Borgou, Collines, Couffo, Donga, Littoral, Mono, Quémé, Plateau, and Zou. The country spans from north to south and a long stretched country in West Africa, located west of Nigeria and east of Togo. It is bordered to the north by Niger and Burkina Faso, in south by the Bight of Benin, in the Gulf of Guinea, that part of the tropical North Atlantic Ocean which is roughly south of West Africa. Benin’s former name, until 1975, was Dahomey. Benin has a population of 11.48 million people (in 2018) [15], and Porto-Novo, a port on an inlet of the Gulf of Guinea, is the nation’s capital city. The largest city and economic capital is Cotonou. Spoken languages are French (official), Fon, and Yoruba [16].

### Selection criteria

We included women who became pregnant after the guideline of ≥ 8 ANC contacts has been endorsed by WHO [3]. In the guideline, ANC models with a minimum of eight contacts are recommended to reduce perinatal mortality and improve women’s experience of care. The 2016 WHO ANC model replaced the previous four-visit FANC model. Accordingly, any woman who could not recall the approximate number of ANC contacts was excluded from the study.

### Outcome variable

ANC was measured dichotomously: < 8 ANC contacts vs. ≥ 8 ANC contacts. The WHO ANC guideline recommendations mapped to the eight recommended contacts and present a summary framework for the 2016 WHO ANC model in support of a positive pregnancy experience [3, 17, 18].

### Explanatory variables

Household wealth quintile**:** principal component analysis (PCA) was used to assign the wealth indicator weights. This procedure assigned scores and standardized the wealth indicator variables such as bicycle, motorcycle/scooter, car/truck, main floor material, main wall material, main roof material, sanitation facilities, water source, radio, television, electricity, refrigerator, cooking fuel, furniture, and number of persons per room. The factor coefficient scores (factor loadings) and z-scores were calculated. For each household, the indicator values were multiplied by the loadings and summed to produce the household’s wealth index value. The standardized z-score was used to disentangle the overall assigned scores to poorest/poorer/middle/richer/richest categories [19, 20]. Furthermore, neighborhood socioeconomic disadvantaged level used items such as rural residence, poorest household wealth level, no formal education, and not working. Using PCA, the standardized z-score was used to disentangle the overall assigned scores to low, medium, and high.

Women’s enlightenment was measured from a list of data elements (education, read newspaper/magazines, listen to radio, and watch television), and decision-making power was measured from a list of data elements (respondent involvement in the decision on how to spend her earnings, decision on her health care, decision on large household purchases, decision on visits to family or relatives, decision on what to do with money husband/partner earns). Using PCA, the factors were distilled into a more generalized set of weights that score “women’s enlightenment” and “decision making power” between 0 and 100. The standardized z-scores were used to disentangle the overall assigned scores to low, medium, and high.

In addition, the following factors were examined: maternal age 15–19/20–24/25–29/30–34/35–39/40–44/45–49; age at first marriage (years): < 18/18–24/>24; family type: monogyny/polygyny; timing to ANC contact initiation: early booking (within 1st trimester)/late booking (after 1st trimester); number of children ever born: 1–3/> 3; birth order: 1st/2nd/3rd/4th/> 4th; preceding birth interval: first birth/< 24 months/24–36 months/> 36 months; religion: Christianity/Islam/Traditional and others; sex of household head: male/female; ever had a terminated pregnancy: yes/no; health insurance coverage: not covered/covered; marital status: not married/currently married or living with a partner/formerly married; wanted pregnancy when became pregnant: then/later/no more; maternal education: no formal education/primary/secondary/tertiary; and place of residence: urban/rural. These factors were included based on previous studies which examined the factors associated with maternal health care services [13, 21].

### Ethical consideration

This study was based on an analysis of population-based dataset available in public domain/online with all identifier information removed. The authors communicated with MEASURE DHS/ICF International, and permission was granted to download and use the data. The DHS project obtained the required ethical approvals from the relevant research ethics committee in Benin, West Africa, before the survey was conducted to ensure that the protocols are in compliance with the U.S. Department of Health and Human Services regulations for the protection of human subjects. Written informed consents were obtained from participants before being recruited in the surveys.

### Statistical analysis

Women’s characteristics were presented using percentages. The collinearity testing approach adopted the correlation method to estimate interdependence between variables. A cutoff of 0.7 was used to examine the multicollinearity known to cause major concerns [22]. Birth interval was retained in the logistic model as it was found to have strong association with birth order. Other significant variables from the bivariate analysis were retained in the model due to lack of multicollinearity. The Survey (“svy”) module was used to adjust for stratification, clustering, and sampling weights to compute the estimates of **≥** 8 ANC contacts across the study variables. Chi-square test was used to examine the association between **≥** 8 ANC contacts and the explanatory variables. All significant variables from the bivariate analysis were included in the multivariable logistic regression model to calculate the adjusted odds ratios (with corresponding 95% CI). Based on the estimation of multivariable logistic regression model, we predicted the probability of **≥** 8 ANC contacts. Thus, $\Pr \left(Y=1|\mathrm{Set}\left[\mathrm{E}=\mathrm{e}\right]\right)=\sum \limits_z{\hat{p}}_{ez}\Pr \left(Z=z\right)$ where Set[E = e] reflects putting all observations to a single exposure level *e*, and Z = z refers to a given set of observed values for the covariate vector Z. Furthermore, ${\hat{p}}_{ez}$ is the predicted probabilities of **≥** 8 ANC contacts for any *E* = e and Z = z. The marginal effects indicate a weighted average over the distribution of the covariates and are equal to estimates got by standardizing to the entire population. As a post-logistic regression test, the exposure *E* is set to the level *e* for all women in the dataset, and the logistic regression coefficients are used to compute predicted probabilities for every woman at their observed covariate pattern and newly exposure value. Because predicted probabilities are computed under the same distribution of *Z*, there is no covariate of the corresponding effect measure estimates [23].

Furthermore, Lorenz curves were used to present socioeconomic inequalities as a plot of cumulative proportion of ≥ 8 ANC contacts among women against cumulative proportion of the population ordered by household wealth quintiles and maternal education. Concentration Index (CI) is positive when the Lorenz curve is below the line of equality indicating the concentration of **≥** 8 ANC contacts concentrates among high socioeconomic groups and vice versa. The urban vs. rural place of residence was used for stratified analyses. In the Lorenz curves, women were ranked according to ascending household wealth-related status to estimate their position in the cumulative distribution of socioeconomic status. Conventionally, when it is applied to binary indicators, the concentration index depends on the mean of the indicator. This would impede cross-factor comparisons because there are substantial differences in means between locations. To address this issue, we used an alternative but related index introduced by Erreygers [24]. Statistical significance was determined at *p* < 0.05. Data analysis was conducted using Stata Version 14 (StataCorp., College Station, TX, USA).

## Results

### Characteristics of women

In total, 1094 women who became pregnant after the policy of **≥** 8 ANC contacts was endorsed were included in the study. The coverage of ≥ 8 ANC contacts was 8.0%; 95% CI 6.5%, 9.7%. Women between 25–29 years (9.5%) and 30–34 years (10.8%) accounted for the highest coverage of **≥** 8 ANC contacts. Women from low neighborhood socioeconomic disadvantaged group reported the highest coverage of about 14.9% of **≥** 8 ANC contacts. In addition, women with low enlightenment (2.5%) and low decision-making power (5.2%) had the least coverage of **≥** 8 ANC contacts. Women who got married at age > 24 years, in monogynous marriage, initiated ANC contacts within first trimester, had 1–3 children, and 1st in birth order or Christians, had the highest coverage of **≥** 8 ANC contacts (13.9%, 9.2%, 14.9%, 9.3%, and 11.8% respectively). Those who had history of terminated pregnancy (12.3%), covered by health insurance (44.4%), and not married (12.5%) had higher coverage of **≥** 8 ANC contacts in Benin. Several maternal characteristics were significantly associated with **≥** 8 ANC contacts (*P* < 0.05) at the bivariate analysis are presented in Table 1.

**Table 1:** Characteristics of women attending ≥ 8 ANC contacts in Benin, West Africa, BDHS 2017–18

Variable	≥ 8 ANC visits		Total	*P* value
	No (92.0%)	Yes (8.0%)		
**Maternal age**				
15–19	116 (95.9)	5 (4.1)	121	0.219
20–24	260 (92.9)	20 (7.1)	280	
25–29	284 (90.5)	30 (9.5)	314	
30–34	181 (89.2)	22 (10.8)	203	
35–39	111 (93.3)	8 (6.7)	119	
40–44	38 (95.0)	2 (5.0)	40	
45–49	17 (100.0)	0 (0.0)	17	
**Neighborhood socioeconomic disadvantaged status**				
Tertile 1 (least disadvantaged)	309 (85.1)	54 (14.9)	363	< 0.001*
Tertile 2	342 (93.4)	24 (6.6)	366	
Tertile 3 (most disadvantaged)	356 (97.5)	9 (2.5)	365	
**Women’s enlightenment level**				
Low	422 (97.5)	11 (2.5)	433	< 0.001*
Medium	291 (91.2)	28 (8.8)	319	
High	315 (92.1)	27 (7.9)	342	
**Decision-making power**				
Low	273 (94.8)	15 (5.2)	288	0.022*
Medium	213 (89.1)	26 (10.9)	239	
High	85 (87.6)	12 (12.4)	97	
**Age at first marriage (years)**				
< 18	377 (94.3)	23 (5.7)	400	0.029*
18–24	521 (91.7)	47 (8.3)	568	
> 24	74 (86.1)	12 (13.9)	86	
**Family type**				
Monogyny	624 (90.8)	63 (9.2)	687	0.017*
Polygyny	326 (95.0)	17 (5.0)	343	
**Timing of ANC initiation**				
Early booking (within 1st trimester)	441 (85.1)	77 (14.9)	518	< 0.001*
Late booking (after 1st trimester)	440 (99.5)	2 (0.5)	442	
**Number of children ever born**				
1–3	565 (90.7)	58 (9.3)	623	0.056
> 3	442 (93.8)	29 (6.2)	471	
**Birth order**				
1st	210 (88.2)	28 (11.8)	238	0.042*
2nd	174 (92.1)	15 (7.9)	189	
3rd	181 (92.3)	15 (7.7)	196	
4th	149 (90.8)	15 (9.2)	164	
> 4th	293 (95.4)	14 (4.6)	307	
**Preceding birth interval**				
First birth	212 (87.6)	30 (12.4)	242	0.002*
< 24 months	114 (99.1)	1 (0.9)	115	
24–36 months	337 (92.3)	28 (7.7)	365	
> 36 months	344 (92.5)	28 (7.5)	372	
**Religion**				
Christianity	457 (88.2)	61 (11.8)	518	< 0.001*
Islam	383 (96.7)	13 (3.3)	396	
Traditional and others	167 (92.8)	13 (7.2)	180	
**Sex of head of household**				
Male	856 (92.2)	72 (7.8)	928	0.575
Female	151 (91.0)	15 (9.0)	166	
**Ever had a terminated pregnancy**				
Yes	93 (87.7)	13 (12.3)	106	0.084
No	914 (92.5)	74 (7.5)	988	
**Health insurance coverage**				
Not covered	1001 (92.3)	84 (7.7)	1085	< 0.001*
Covered	5 (55.6)	4 (44.4)	9	
**Marital status**				
Not married	35 (87.5)	5 (12.5)	40	0.523
Currently married/living with a partner	955 (92.2)	81 (7.8)	1036	
Formerly married	17 (94.4)	1 (5.6)	18	
**Currently residing with husband/partner**				
Living together	812 (92.2)	69 (7.8)	881	0.969
Staying elsewhere	143 (92.3)	12 (7.7)	155	
**Wanted pregnancy when became pregnant**				
Then	731 (92.1)	63 (7.9)	794	0.992
Later	210 (92.1)	18 (7.9)	228	
No more	66 (91.7)	6 (8.3)	72	

*Significant at *p* < 0.05

### Factors associated with ≥ 8 ANC contacts (multivariable logistic regression)

The results of timing to antenatal care initiation showed that women who had late booking (after 1st trimester) had 97% reduction in ≥ 8 ANC contacts compared with women who initiated ANC contacts within the first trimester (AOR = 0.03; 95% CI 0.00, 0.21). In addition, women with medium or high enlightenment were 4.55 and 5.49 times as likely to have ≥ 8 ANC contacts, compared with women having low enlightenment (AOR = 4.55; 95% CI 1.41, 14.69 and AOR = 5.49; 95% CI 1.77, 17.00) Table 2.

**Table 2:** Logistic regression of the factors associated with ≥ 8 ANC contacts among women of reproductive age in Benin, West Africa, BDHS 2017–2018

Variable	AOR	95% CI	*P*
**Timing to ANC contacts initiation**			
Early booking (within 1st trimester)	1.00		
Late booking (after 1st trimester)	0.03	0.00–0.21	0.001*
**Women’s enlightenment level**			
Low	1.00		
Medium	4.55	1.41–14.69	0.011*
High	5.49	1.77–17.00	0.003*

Model adjusted for neighborhood socioeconomic disadvantaged level, decision-making power, age at 1st marriage, family type, preceding birth interval, religion, and health insurance coverage

*Significant at *p* < 0.05

### Marginal effects of the factors associated with ≥ 8 ANC contacts

In Table 3, marginal effect analysis was conducted to decipher the effects of the factors associated with ≥ 8 ANC contacts. From the predictive marginal effects results, assuming the distribution of all factors remained the same among women, but every woman had low neighborhood socioeconomic disadvantaged status, we would expect 11% of ≥ 8 ANC contacts. If every woman had high maternal enlightenment or high decision-making power, we would expect 11% and 8% of ≥ 8 ANC contacts. If instead the distribution of neighborhood socioeconomic disadvantaged status, maternal enlightenment, and decision-making power were as observed and other covariates remained the same among women, but no woman had first marriage before age 25 years or had monogynous family life, we would expect about 10% and 9% of ≥ 8 ANC contacts respectively. Furthermore, if instead the spread of the aforementioned variables were as observed and other covariates remained equal among women, but every woman initiated ANC contacts within 1st trimester were Christians or had health insurance coverage, we would expect 13%, 9%, and 16% of ≥ 8 ANC contacts respectively. In Table 3, we practically obtained the predictive marginal effects of the factors associated with ≥ 8 ANC contacts.

**Table 3:** Marginal effect of the factors associated with the frequency of ANC visits among women of reproductive age in Benin, West Africa, BDHS 2017–2018

Variable	Marginal effect	95% CI	*P*
**Neighborhood socioeconomic disadvantaged status**			
Tertile 1 (least disadvantaged)	0.11	0.07–0.15	< 0.001*
Tertile 2	0.07	0.04–0.11	< 0.001*
Tertile 3 (most disadvantaged)	0.05	0.01–0.09	0.018*
**Maternal enlightenment level**			
Low	0.03	0.00–0.05	0.039*
Medium	0.10	0.05–0.14	< 0.001*
High	0.11	0.07–0.15	< 0.001*
**Decision-making power**			
Low	0.08	0.04–0.12	< 0.001*
Medium	0.09	0.06–0.12	< 0.001*
High	0.08	0.04–0.13	0.001*
**Age at first marriage (years)**			
< 18	0.08	0.04–0.13	< 0.001*
18–24	0.08	0.05–0.11	< 0.001*
> 24	0.10	0.04–0.17	0.002*
**Family type**			
Monogyny	0.09	0.06–0.12	< 0.001*
Polygyny	0.07	0.03–0.11	< 0.001*
**Timing to ANC visits initiation**			
Early booking (within 1st trimester)	0.13	0.10–0.17	< 0.001*
Late booking (after 1st trimester)	0.01	− 0.01–0.02	0.315
**Preceding birth interval**			
First birth	0.10	0.04–0.16	0.001*
< 24 months	0.02	− 0.02–0.05	0.310
24–36 months	0.09	0.05–0.12	< 0.001*
> 36 months	0.10	0.06–0.13	< 0.001*
**Religion**			
Christianity	0.09	0.07–0.12	< 0.001*
Islam	0.06	0.02–0.10	0.004*
Traditional and others	0.08	0.02–0.14	0.010*
**Health insurance coverage**			
Not covered	0.08	0.06–0.10	< 0.001*
Covered	0.16	0.02–0.30	0.029*

*Significant at *p* < 0.05; McFadden’s R-squared for model fitness = 0.28

### Socioeconomic inequalities (Lorenz curve and concentration index)

**Fig. 1:**
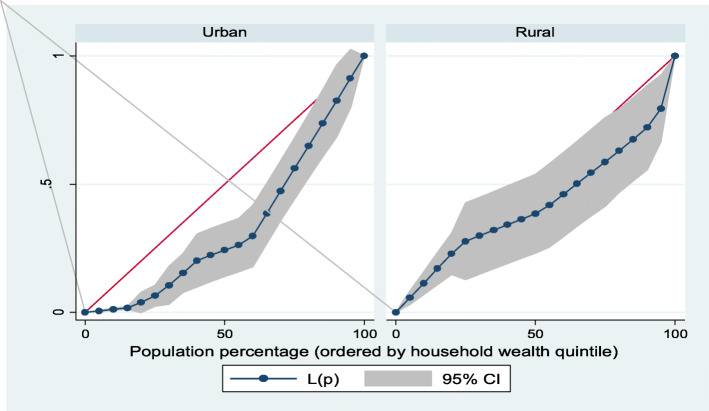
Urban-rural differential in household wealth-related inequalities of ≥ 8 ANC contacts

**Fig. 2:**
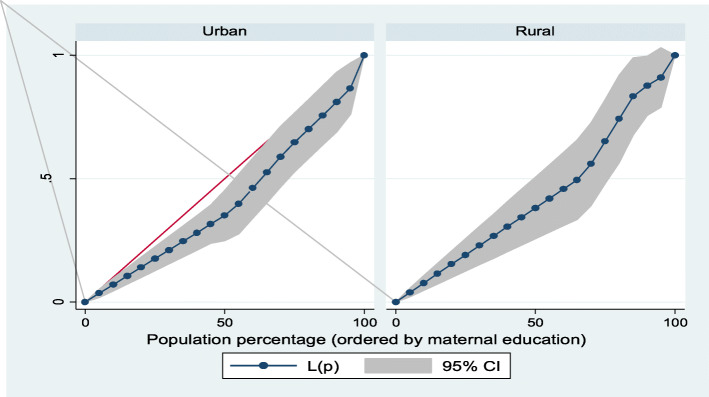
Urban-rural differential in maternal education inequalities of ≥ 8 ANC contacts

**Fig. 3:**
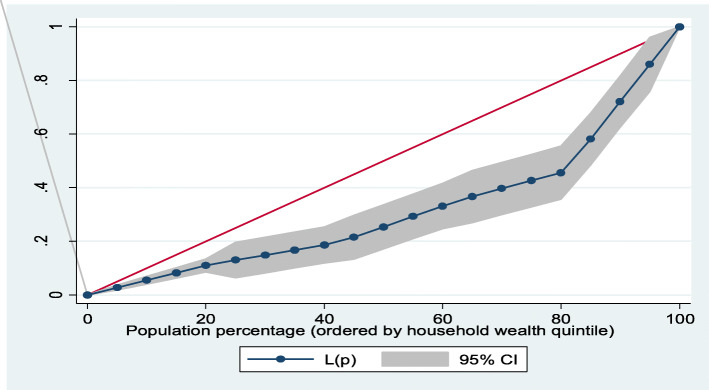
Overall household wealth-related inequalities of ≥ 8 ANC contacts

**Fig. 4:**
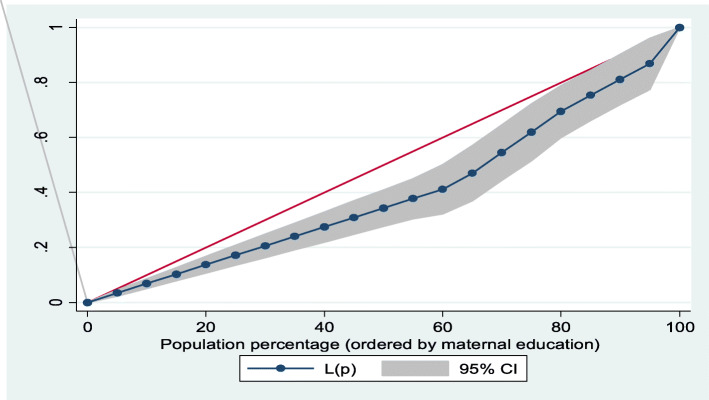
Overall maternal education inequalities of ≥ 8 ANC contacts

Figures 1, 2, 3, 4 showed the household wealth-related and maternal education inequalities for women of reproductive age who had ≥ 8 ANC contacts in Benin, West Africa. The farther the Lorenz curves sags away from the line of equality, the greater the degree of inequalities. The curves revealed that women from higher wealth group and maternal education had higher ≥ 8 ANC contacts.

In Table 4, we presented the coverage of ≥ 8 ANC contacts across household wealth quintile and maternal education. Overall, the richest women and higher maternal education had the highest coverage of ≥ 8 ANC contacts. Using Concentration Index, we quantified the degree of wealth-related and maternal education inequalities in ≥ 8 ANC contacts. The outcome (≥ 8 ANC contacts) was significantly more in the higher household wealth groups and maternal education, specifically among the urban women and total sample: Household wealth-related factor: urban women—Conc. Index = 0.33; *p* < 0.001; total sample—Conc. Index = 0.37; *p* < 0.001. Maternal education: urban women—Conc. Index = 0.18; *p* = 0.006; total sample—Conc. Index = 0.21; *p* < 0.001).

**Table 4:** Prevalence and Concentration Index of ≥ 8 ANC contacts by household wealth quintile and maternal education, BDHS, 2017-18

Variable	Urban	Rural	Total
**Household wealth quintile (%)**			
Poorest	1.5	5.6	4.4
Poorer	6.8	2.1	3.0
Middle	12.5	4.1	6.2
Richer	5.1	4.4	4.7
Richest	22.7	20.0	22.2
Overall	12.9	4.9	8.0
Concentration Index	0.33	0.16	0.37
SE	0.07	0.10	0.06
*P* value^**α**^	< 0.001*	0.087	< 0.001*
Urban-rural comparison			
z-stat	− 1.46		
CI difference	− 0.17		
*P* value^**β**^	0.143		
**Maternal education** (%)			
No education	9.1	3.7	5.5
Primary	16.5	8.9	11.9
Secondary	14.2	3.3	9.1
Tertiary	50.0	40.0	47.1
Overall	12.9	4.9	8.0
Concentration Index	0.18	0.15	0.21
SE	0.07	0.08	0.05
*P* value^**α**^	0.006*	0.067	< 0.001*
Urban-rural comparison			
z-stat	− 0.34		
CI difference	− 0.03		
*P* value^**β**^	0.734		

*P* value^α^ and *P* value^β^ were obtained using the Concentration Index for overall inequalities across socioeconomic groups and measuring rural vs. urban differences respectively

*SE* standard error

*Significant at *p* < 0.05

## Discussion

About 3 years (since November 2016) after the publication and dissemination of ≥ 8 ANC contacts guideline, this survey is the foremost to select respondents and gauges the status and extent of in-country utilization and adaptation, and whether the recommendation in the guideline has been implemented or influenced policy decisions. The recommendation requires that the first ANC contact should take place in the first 12 weeks of gestation (within first trimester of the pregnancy); thereafter, two other contacts are scheduled to take place during the 20th and 26th weeks of gestation (that is, during the second trimester of the pregnancy), and the remaining five contacts are scheduled to place during the third trimester (precisely at 30th, 34th, 36th, 38th, and 40th weeks of gestation) [25].

The results of this study revealed that after about 3 years of the launch of WHO minimum of 8 ANC contacts model, less than one tenth (only 8.0%) of women in Benin had at least 8 ANC contacts with the health care providers throughout the period of their pregnancy. In a previous study, which examined the coverage of minimum 4 ANC visits in Benin, the findings from 2006 and 2012 data showed the coverage were about 61.0% and higher than the current minimum of 8 ANC contacts [13]. This result clearly showed that the new WHO 8 ANC contacts model is yet to be institutionalized or accepted among women of childbearing age in Benin Republic. It is most likely that supply-side and demand-side factors could be responsible for this low minimum of 8 ANC contacts uptake. For example, lack of behavior change communication/awareness, poor health care system, or lack of buy-in by stakeholders in health system including the providers can be linked to this staggering outcome.

In the marginal effects model, women from the least socioeconomic disadvantaged neighborhood were found to have had higher predictive value of ≥ 8 ANC contacts. This can be viewed in the light that women who live in an economic advantaged neighborhood may be well-off, more enlightened, and have more access to health facility than those living in economically disadvantaged neighborhood. The pro-rich neighborhood is commonly where the members of richest wealth quintile, educated, and the affluent members of a given society dwell. These areas are commonly equipped with the best of health care, technology, and education compared with the poor or socioeconomically most disadvantaged areas. This phenomenon abounds in most parts of SSA countries. The reason for the inability to have maximum ANC contacts has over the years been linked to financial constraints, poor access to health facility, lack of decision-making power as regard to reproductive health matters, proximity to health facility, and so forth [12, 26–28]. These factors that are linked to low frequency of ANC contacts should be of great concern to policy makers and funding agencies. Some studies have acknowledged lack of knowledge of the importance of ANC contacts, ignorance of the time to start ANC contacts, as reasons for underutilization of ANC in general [12, 27, 29]. This finding corroborates with the present results from the analysis of socioeconomic inequalities, where the educated women and those from households with high wealth status had greater uptake of ANC contacts as recommended in the new guideline.

The enlightenment level of pregnant women was also found to be correlated with ≥ 8 ANC contacts. Improved enlightenment levels usually result from exposure to educational materials, listening to news, watching television, reading newspapers, and passing through a formal education. This may be related to the knowledge of the importance of registering for ANC and also completing at least 8 ANC contacts. These factors have previously been reported to positively influence optimal utilization of ANC among women in Benin [30] and in rural Malawi [31]. Moreover, we observed that high decision-making power is a factor that increased at least 8 ANC contacts. This factor could result from increased autonomy for the women, more access to health information, and an enhanced economic and monetary status [12]. Therefore, it is important that the girl child should be educated up to post-secondary school level and empowered for greater independence such that the issues concerning her health needs especially the reproductive aspect will be decisively taken by her. In addition, more communication through using mass media providing information about the importance of this new ANC model would be helpful in facilitating behavior change [32]. The multivariable logit model shows that women who were highly or moderately enlightened were more likely to have 8 or more antenatal care contacts. Education or enlightenment has been reported to be a protective factor that enhances women’s utilization of health care services as well as reproductive health decision-making [33, 34].

Women who married after age 24 years had higher predictive values of at least 8 ANC contacts. This finding is in agreement with the report of a previous study where the majority of the pregnant women who had good utilization of ANC contacts had their marriage after adolescent age [35]. The explanations could be that the delay of timing to marriage may be due to spending more time in educational institution or career development [36], which is a measure of improving women’s empowerment. These folks could have better knowledge of the benefits of optimal ANC contacts during pregnancy which is in line with reports from previous studies [35, 37]. Education is one of the major ways to empower women. It provides them with higher assurance and competence to make decisions in the use of available modern health care services for themselves and their children [33].

In addition, women who initiated ANC early or at the first trimester of pregnancy had higher predictive value of ≥ 8 ANC contacts. Also, in the logit model, it was found that late booking for ANC had large reduction in the odds of having at least 8 ANC contacts during pregnancy. Similarly, the findings from previous studies showed that early booking for ANC would result in optimal number of ANC contacts during pregnancy [38]. Furthermore, women who had health insurance coverage, monogynous family, long preceding birth interval or first childbirth, and of Christian religious belief had increased predictive values to the uptake of a minimum of 8 ANC contacts.

The findings from the socioeconomic inequalities of ≥ 8 ANC contacts revealed that women from non-poor households and the educated women had higher uptake of ≥ 8 ANC contacts, especially in the urban residence. This is similar to previous results obtained for the utilization of maternal health care services in Benin [13]. The SDG-3 is known to support reduction in inequalities and ensure health for all populations. Therefore, beyond the utilization of ≥ 8 ANC to reach the most disadvantaged group of women, the uptake of maternal care services must be considered to achieve the set goals. Strengthening health care programs and policies can enhance optimal ANC contacts uptake among women of reproductive age, as well as improve the utilization of maternal care services, particularly for the uneducated, low household wealth quintiles, and those who live in hard-to-reach rural residence.

### Strengths and limitations

This is the foremost nationwide analyses that explore the uptake of at least 8 ANC contacts in Benin and as such could serve as a benchmark and stimulus for further nationwide studies on related subject matter. Another strength is the use of current nationally representative datasets which makes the findings of the study generalizable to women of reproductive age in Benin. However, this analysis has some drawbacks. Prominently, the analyses utilized cross-sectional data; hence, only associations and no causal relationships could be established. Moreover, our inability to measure sources of demand-side unobserved heterogeneity across the secondary data might have biased our estimates. The unavailability of relevant variables other than those collected was a major limitation in the DHS data. Furthermore, recall bias could have occurred due to the self-reported number of ANC contacts.

## Conclusion

Analysis of the BDHS showed low coverage of ≥ 8 ANC contacts in Benin. In addition, women’s enlightenment, early ANC initiation, and socioeconomic inequalities determined the coverage of ≥ 8 ANC contacts. Effective implementation of the new ANC contacts guidelines will essentially require reorganization of care and redistribution of health care resources. The potential low implementation could be resolved by improving behavior change communication among women of reproductive age to improve their understanding of the importance of the new model of care. Enlightening women and providing support for early initiation of ANC contacts and increasing equity-focused efforts will also help to improve the uptake of the minimum 8 ANC contacts.

## Data Availability

Data for this study were sourced from Demographic and Health surveys (DHS) and available here: http://dhsprogram.com/data/available-datasets.cfm.

## Abbreviations

ANC: Antenatal care
FANC: Focused antenatal care
MDGs: Millennium Development Goals
SDGs: Sustainable Development Goals
SSA: Sub-Saharan Africa
UN: United Nations
WHO: World Health Organization
